# Native Valve Candida metapsilosis Endocarditis Following a Ruptured Appendix: A Case Report

**DOI:** 10.7759/cureus.21178

**Published:** 2022-01-12

**Authors:** Koushik Sanku, Dima Youssef

**Affiliations:** 1 Department of Internal Medicine, East Tennessee State University Quillen College of Medicine, Johnson City, USA; 2 Department of Internal Medicine, Division of Infectious Diseases, East Tennessee State University Quillen College of Medicine, Johnson City, USA

**Keywords:** echocardiography, appendix rupture, metapsilosis, parapsilosis, immunocompetent, invasive fungal disease, aortic valve replacement, candida endocarditis, infective endocarditis, native valve

## Abstract

*Candida parapsilosis* complex has been further divided into *C. parapsilosis*, *C. orthopsilosis*, and *C. metapsilosis*. *C. metapsilosis* is considered to be the least virulent fungi of the complex. Candida endocarditis is uncommon but is associated with a very high mortality rate. Prosthetic or previously damaged valves act as common targets, but native, structurally normal valves are seldom affected.

We hereby present a case of *Candida metapsilosis* endocarditis involving a native aortic valve in an immunocompetent 55-year-old male who was successfully treated with surgical valve replacement and antifungal therapy.

## Introduction

Candida is an uncommon cause of infective endocarditis (IE) accounting for about 1-2% of all cases of IE but can be associated with a remarkably high mortality rate [1]. Candida species are normal flora on human skin, the gastrointestinal tract, and the lower genitourinary tract [2,3]. 

Candida usually affects damaged heart valves or hardware, like prosthetic heart valves (2%-10%), pacemakers (4.5%), implantable cardioverter defibrillators, and ventricular assist devices (35%-39%) by forming biofilms [2]. However native valve endocarditis is seldom reported but may be seen in presence of other concurrent risk factors like immunosuppression, intravenous drug use, neutropenia, diabetes mellitus, chronic indwelling central venous catheters, parenteral nutrition, previous abdominal or cardiac surgery [1,2]. 

We hereby presented a case of native valve endocarditis caused by *Candida metapsilosis* following an appendiceal rupture. 

## Case presentation

A 55-year-old male patient presented to the emergency department with a five-day history of worsening right lower quadrant abdominal pain, 8/10 in severity associated with fever, nausea, and constipation. Physical examination revealed severe right lower quadrant tenderness. He had a white blood cell (WBC) count of 13.3 x 10^9^/L and abdominal imaging was positive for a ruptured appendix and right paracolic abscess. The patient was treated conservatively with intravenous (IV) piperacillin-tazobactam and percutaneous drain placement. His drain cultures grew *E. coli* susceptible to piperacillin-tazobactam. He remained afebrile but his leukocytosis continued to worsen over the next few days. Repeat imaging revealed multiple abscesses in the right abdomen along with a large right-sided pleural effusion. A second percutaneous abdominal drain was placed, and an ultrasound-guided thoracentesis was done to drain the right pleural effusion. Subsequently, his WBC count trended down, blood cultures and fluid cultures remained negative, and the patient was discharged on a 10-day course of IV ertapenem 1 g and oral fluconazole 200 mg once daily. An echocardiogram done prior to discharge showed no diastolic dysfunction or valvular abnormalities. 

The patient failed to follow up with the primary care physician on completing the antibiotic course before eventually presenting to the clinic two months later complaining of episodic fevers, chills, diaphoresis, generalized fatigue, and diarrhea. 

Investigations 

**Figure 1:**
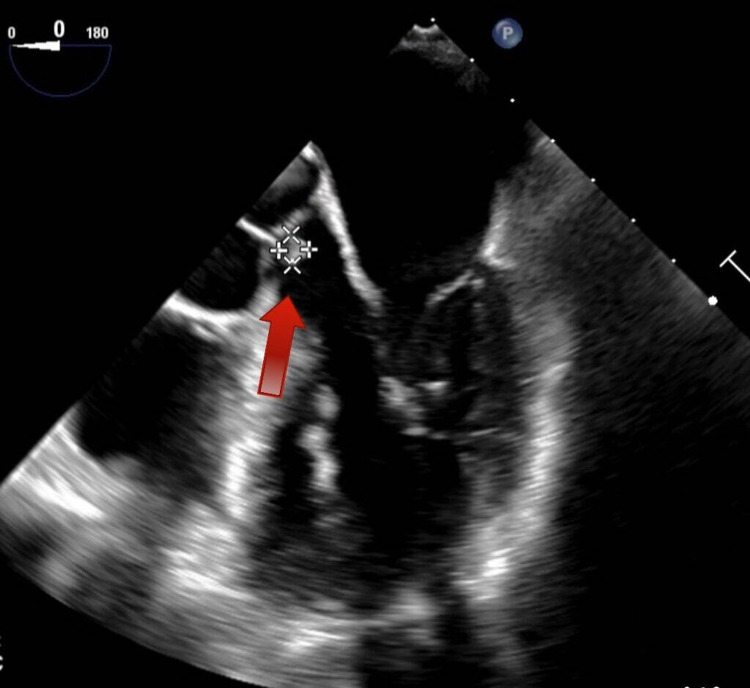
Transesophageal echocardiogram (TEE) showing an echogenic mass (red arrow) that represents the septic vegetation attached to the aortic valve cusp

**Figure 2:**
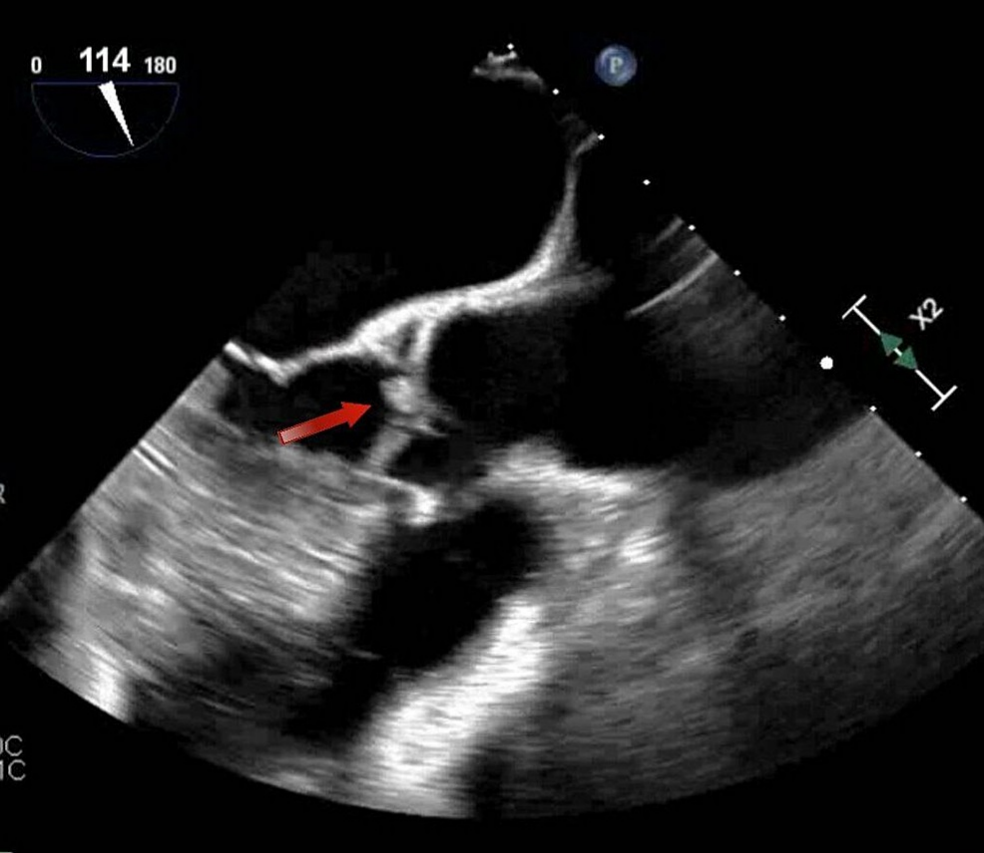
Transesophageal echocardiogram (TEE) showing vegetation attached on the ventricular side of aortic valve

The patient's labs, CT (Computed Tomography) of chest and abdomen were unremarkable, but his blood cultures were positive for *Candida metapsilosis*. He was admitted to the hospital and was started on IV anidulafungin 200 mg once daily. Transthoracic echocardiogram (TTE) showed an ejection fraction (EF) of 60-65% with a suspicious mobile echogenic area noted on the aortic valve. A subsequent transesophageal echocardiogram (TEE) showed aortic valve vegetation on the ventricular side with mild aortic regurgitation (Figures 1, 2) and mild mitral regurgitation with moderate calcified mixed atheromatous aortic plaque. The patient denied any previous intravenous drug use. The patient did not have any other signs of infectious endocarditis. Repeat blood cultures 48 hours later also grew Candida despite being on antifungal therapy. 

Treatment 

Thus, antibiotic therapy was switched to IV liposomal amphotericin B 3mg/kg/dose once daily and oral flucytosine 1500 mg every six hours based on sensitivities. However, the patient was unable to clear the infection, and a total of six sets of blood cultures repeated every two days continued to grow *Candida metapsilosis*. Eventually, cardiothoracic surgery was consulted, and the patient underwent aortic valve replacement. Postoperatively he was started on IV ceftriaxone while continuing amphotericin B and flucytosine. Blood cultures obtained during the postoperative period remained negative but, cultures from the resected valve grew *Candida metapsilosis*, confirming it as a source of the patient's persistent candidemia. He remained asymptomatic and clinically stable over the next few days and was subsequently discharged on chronic suppressive anti-fungal therapy. 

Outcome and follow-up 

The patient was continued on IV amphotericin B and flucytosine for six weeks following which he was transitioned to a lifetime course of daily oral fluconazole. On subsequent follow-up visits, the patient remained free of any disease recurrence and was tolerating oral fluconazole well. 

## Discussion

We report an unusual case of native aortic valve endocarditis due to *Candida metapsilosis* in an immunocompetent individual. Modified Duke’s criteria were used for the diagnosis of infective endocarditis as the patient met two major criteria, i.e., persistent candidemia on blood cultures and valvular vegetations on the echocardiogram [4].

Invasive Candida infections are often caused by *C. albicans* followed by *C. parapsilosis* complex, *C. gabrata*, and *C. tropicalis* [1]. *C. parapsilosis* complex is further subdivided by molecular studies into *C. parapsilosis*, *C. orthopsilosis*, and *C. metapsilosis* [3,5]. Although *Candida parapsilosis *complex is less virulent than *C. albicans*, *C. parapsilosis* complex has high rates of resistance to echinocandins and some azoles, making this infection difficult to treat [1,3].

*C. parapsilosis* is a normal human commensal and is often isolated from the subungual space of human hands [3]. It has also been isolated from gastrointestinal and genitourinary samples thus indicating its ability to colonize the human body [2,3]. However, invasive disease caused by *C. parapsilosis* can occur without prior colonization and is often transmitted in a healthcare setting via sources such as medical devices or fluids, the hands of health care workers, prosthetic devices, and catheters [3]. Most important predisposing factors for *C. parapsilosis* endocarditis include the presence of pre-existing valvular disease, implanted prosthetic valves or devices, immunosuppression, IV drug use, neutropenia, chronic indwelling central venous catheters, parenteral nutrition, diabetes mellitus, and previous abdominal surgery [1-3]. Although we have no clear way of proving the source of infection, our patient’s recent history of appendiceal rupture makes the GI tract a likely source. As the integrity of the bowel is disrupted in case of a ruptured appendix, colonized organisms like Candida could potentially leak into the bloodstream resulting in the candidemia we saw in our patient. The nosocomial spread could be another likely explanation, given the patient’s prior hospital stay. The cardiac tissue most commonly infected is the aortic valve [3]. Our patient had septic vegetation involving the cusp of the aortic valve resulting in aortic regurgitation. Other areas that can be involved in the order of decreasing frequency include the mitral valve, tricuspid valve, ventricular wall, and pulmonary valve [3].

Any patient suspected of IE by clinical criteria should be screened using transthoracic echocardiography (TTE). TTE has a sensitivity that ranges between 40-63%. However, if TTE is negative or equivocal and the clinical suspicion for endocarditis is high, transesophageal echocardiography (TEE) should be performed as it has a much higher sensitivity of 90-100%. TEE also has a high negative predictive value of 86-97%, and thus a negative test is highly reliable to rule out IE. However, it’s important to make a clinical correlation as vegetations may not develop in the preliminary stages of the disease. Therefore in such clinical scenarios, it is reasonable to perform a repeat TEE about seven to 10 days later to allow for potential vegetations to become more apparent. A repeated negative study often always rules out the diagnosis of endocarditis [6].

Recommended initial treatment for native valve endocarditis includes liposomal amphotericin B 3-5 mg/kg daily with or without flucytosine 25 mg/kg four times daily. High-dose echinocandin therapy (with caspofungin 150 mg daily or micafungin 150 mg daily, or anidulafungin 200 mg daily) can also be used as a reasonable alternative for initial therapy. Therapy can be deescalated to fluconazole 400 to 800 mg daily provided that the isolated fungi are susceptible, patients are clinically stable, and have cleared Candida from their bloodstream [7]. Valve replacement is often recommended, as a combined approach with surgical replacement of infected valves along with aggressive antifungal therapy is associated with lower mortality rates and decreased disease recurrence [3,7]. However, in patients who are not ideal candidates for valve replacement, chronic suppressive therapy with oral fluconazole 400-800 mg per day can be used as a reasonable alternative [7]. Although there is very little evidence, chronic suppressive antifungal therapy can be used in all patients including those undergoing surgery to decrease the risk of recurrence [7].

## Conclusions

Fungal endocarditis is classically believed to affect prosthetic or damaged heart valves, and thus its diagnosis is often delayed or missed in patients with structurally normal valves. Our case adds to the evidence supporting the possibility of native valve fungal endocarditis. *Candida metapsilosis* is a newly isolated member of the *Candida parapsilosis* family. Although considered to be less virulent, it can be notorious to treat owing to its higher rates of resistance to echinocandins. Although there have been several case reports suggesting a non-surgical approach for the treatment of Candida endocarditis, based on our case and literature review, we support the approach of combining early surgery with aggressive antifungal therapy to be a superior treatment modality.
